# Understanding the interplay between psoriatic arthritis and gout: “Psout”

**DOI:** 10.1007/s00296-024-05729-8

**Published:** 2024-10-23

**Authors:** Alaa Sherri, Mohamad Mahdi Mortada, Joanna Makowska, Milena Sokolowska, Anna Lewandowska‐Polak

**Affiliations:** 1 Department of Rheumatology, Medical University of Lodz, Lodz, Poland; 2 Department of Immunology and Allergy, Medical University of Lodz, Lodz, Poland; 3 Department of Immune Metabolism, Swiss Institute of Asthma and Allergy Research (SIAF), Davos, Switzerland

**Keywords:** Psoriatic arthritis, Psout, Gout, Serum uric acid, Urate crystals, Hyperuricemia

## Abstract

The interplay between Psoriatic arthritis and Gout is a current diagnostic challenge faced by many physicians and researchers. We aimed at reviewing the coexistence of gout and its features such as hyperuricemia and deposition of monosodium urate crystals in patients with psoriatic arthritis (PsA). We also focused on a brief presentation of the pathophysiology underneath the interplay between PsA and gout, and ultimately on recommendation of approaches for the differential diagnosis. The literature search for this narrative review was conducted using PubMed and Medline and after retrieving and screening the references, articles were selected according to the inclusion and exclusion criteria. Part of the assessed studies reported the coexistence of PsA and gout (Psout) and its association with several clinical outcomes among affected patients. Other studies stressed incidences of misdiagnosis of gout with PsA and vice versa. Additionally, the presence of hyperuricemia in PsA patients could interfere with the patient’s characteristics and outcomes of their treatment. Further research on the assessment and clinical course of Psout is required to develop an official protocol for its diagnosis and treatment.

**Supplementary Information:**

The online version contains supplementary material available at 10.1007/s00296-024-05729-8.

## Introduction

Psoriatic arthritis (PsA) is a spondyloarthropathy with a high genetic predisposition [1]. PsA combines the aspects of two opposing manifestations: autoinflammation and autoimmunity [2]. Its heterogeneity explains its characteristic features like psoriasis (PsO), synovitis, enthesitis, spondylitis, and extra-articular manifestations [1–3]. The combination of collecting medical history, physical examination, and appropriate laboratory and imaging tests helped in developing several classification criteria including the CASPAR criteria (classification criteria for psoriatic arthritis) [3, 4]. These criteria are commonly used to diagnose PsA according to its specific features and aid in differentiating it from other inflammatory arthritides, rheumatoid arthritis, and crystal arthropathies with high sensitivity and specificity [5]. However, the issue of interplay and coexistence between PsA and gout is very common and may question the accuracy and precision of recently used classification criteria. Just like the possible co-existence of systemic lupus erythematosus (SLE) and rheumatoid arthritis (RA) in a condition known as (Rhupus) syndrome, the interplay between PsA/PsO and gout is being investigated [6, 7]. Felten et. al presented a case of a 40 year-old man showing clinical and radiological aspects of both PsA and gout, and they proposed the term Psout to describe the interplay between PsA and Gout [7]. This case indicates that the current diagnostic dilemma is whether the patient has gout, PsO, or both and leads to the proposal of the term Psout to describe the interplay between PsA and Gout.[7]. To understand the background of this interplay, researchers have been trying to identify mechanisms behind Psout on molecular and clinical levels. As a result, the most supported pathophysiology mechanism has been linked to the effect of uric acid and monosodium urate crystals deposition (MSU) [7, 8]. This effect raises some challenges among rheumatologists and clinicians especially when features of both gout and PsA flares are found among different patients, especially since synovial fluid analysis is not present at the point of care situations [5, 6]. Although we can find several published studies on the prevalence of hyperuricemia among psoriatic patients and its impact on patient comorbidity and treatment profile, only few articles describe the risk of hyperuricemia and its clinical outcomes in PsA [7]. Moreover, there is a need to acknowledge subgroups of patients that have Psout characteristics for further research and better differentiation among PsA and gout patients.

The main aim of this work is to comprehensively review the coexistence of gout and the prevalence of gout features such as hyperuricemia and deposition of monosodium urate crystals in PsA patients. The secondary aims are (i) to briefly summarize the current views on pathophysiology underneath the interplay between PsA and gout, (ii) to present the characteristics of patients with Psout and (iii) to review the recommended approaches for differential diagnosis.

### Search strategy and methods

**Fig. 1:**
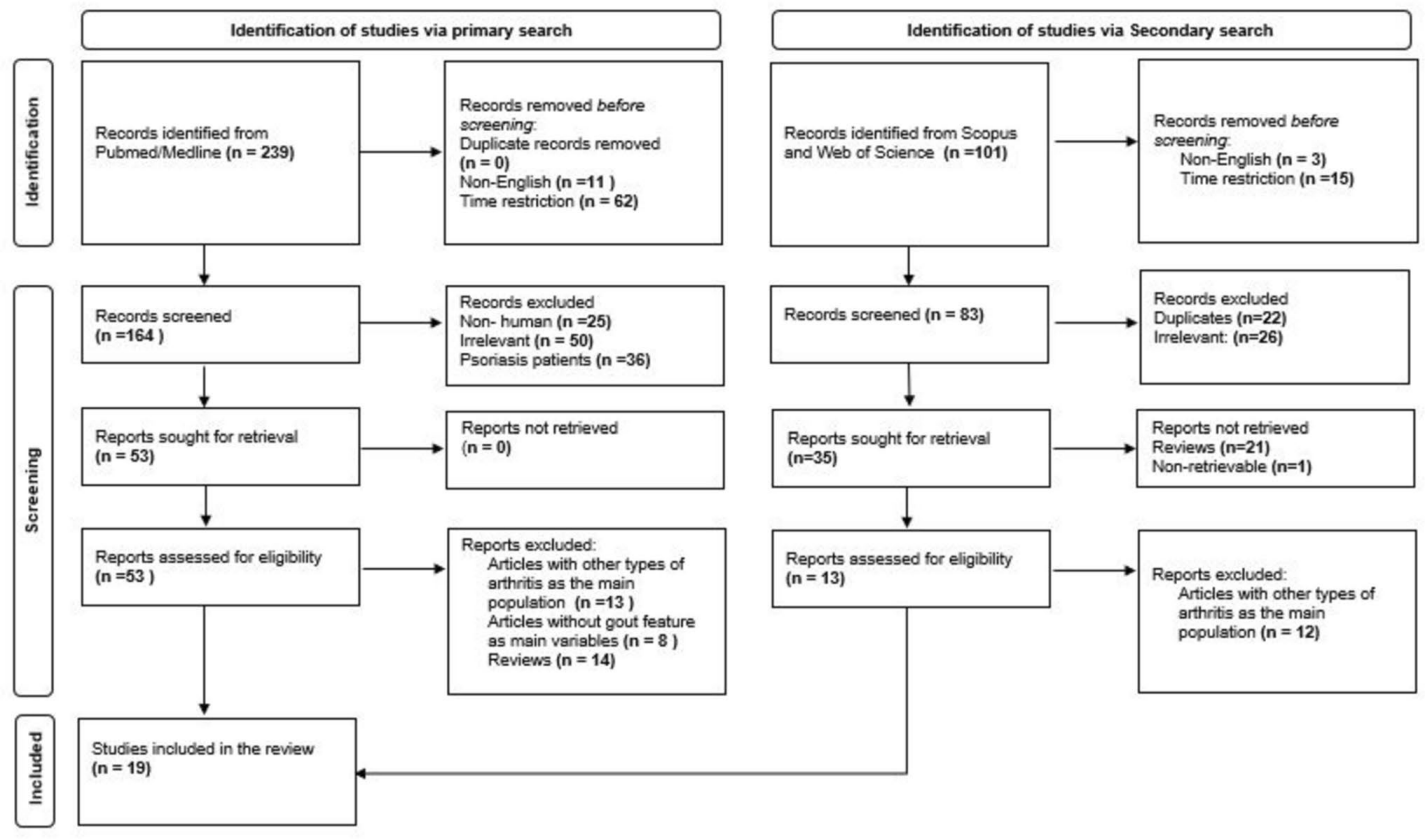
Flowchart showing the criteria used in articles selection

To understand the background of this topic a PubMed/Medline search was done to identify articles and grey literature defining Psout, its background, patients’ characteristics, and pathophysiology. Then, a detailed literature search was conducted to identify studies reporting the prevalence of gout and gout features like hyperuricemia and monosodium urate crystals (MSU) among PsA patients or the interplay between gout and PsA. The comprehensive search was conducted in PubMed/Medline for articles up to January 2024 by two reviewers (AS) and (MMM). The search terms were identified through the PECO strategy as shown in Table 1. The process of article selection started by screening titles and abstracts and then the whole text for eligible articles. Articles were selected according to the inclusion criteria: (1) English articles, (2) Time restriction from 1998 till 2024, (3) Original articles (randomized clinical trials, observational studies, case reports, and meta-analysis), (4) PsA or Psout as main study population, (5) Studies that show features of gout in PsA as main variables. Thus, all non-English, irrelevant to the topic, studies with PsO patients only as the main sample population, and articles before 1998 were excluded. Then, a secondary literature search was initiated on Web of Science and Scopus databases to maximize available data and consider all relevant literature. The same search terms were used in both search queries (Psoriatic arthritis, Gout, hyperuricemia, serum uric acid, and urate crystals), and the same inclusion and exclusion criteria were also applied to obtain one extra included article as shown in Fig. 1.

**Table 1:** PubMed/Medline PECO search strategy using MeSH keywords

	PICO strategy	MeSH terms and keywords
Population (P)	Psoriatic arthritis patients, PSOUT, gout	#1 Psoriatic arthritis [MeSH Terms] #2 Psout #3 Gout [MeSH Terms] #4: #1 OR #2 OR #3
Exposure (E)	Prevalence of gout, serum uric acid levels, and synovial fluid monosodium urate crystals in psoriatic arthritis patients	#5 Serum uric acid #6 urate crystals #7: #5 OR #6
Compare (C)	Psoriatic arthritis with normal uric acid or other arthritis patients	
Outcomes (O)	-Hyperuricemia in patients with psoriatic arthritis or detection of monosodium urate crystals in synovial fluid of psoriatic arthritis patients -Psout caused by hyperuricemia and monosodium urate crystals - Gout misdiagnosed as psoriatic arthritis	#8 Hyperuricemia and psoriatic arthritis #9 Uric acid and psoriatic arthritis #10 Urate crystals and psoriatic arthritis #11 Gout AND Psoriatic arthritis AND misdiagnosis #12: #8 OR #9 OR #10 OR #11
Combined Search strategy	#4 AND #7 OR #12

### Psoriatic arthritis and gout

The American College of Rheumatology aimed to develop a consensus statement on the definition of gout due to the lack of a standardized description. As a result, monosodium urate crystals and hyperuricemia were agreed on as the main components of gout [9]. The importance of hyperuricemia and measurement of serum uric acid among PsA patients was covered in recent studies, and the diagnostic index of hyperuricemia (HUC) compared to other parameters like ultrasound was also investigated [10]. The value of HUC showed more importance especially since many studies displayed it as an independent comorbidity among psoriatic patients developing psoriatic arthropathy. It is also important to understand which of the subtypes of psoriatic disease (psoriasis vulgaris or psoriatic arthritis) are more prone to Psout or HUC in general. In a study by Tsuruta et. al, PsA patients showed a higher frequency of HUC (22%) compared with patients with psoriasis vulgaris (9%), and this association was also confirmed by logistic regression analysis [11]. To identify Psout among PsA patients, it is important to distinguish two subtypes of subjects with PsA: “Hyperuricemia and Normouricemia PsA patients” especially since gout features appear at later stages of PsA.

The studies that show the prevalence of hyperuricemia among PsA patients as the primary sample population are summarized in Table 2. In a study by Al Johani et. al, the prevalence of HUC accounted for 31.9% compared to patients with normal uric acid. Moreover, the patients with HUC showed differences in characteristics compared with normal-uricemic (NUC) patients like longer disease duration of psoriatic arthritis (p < 0.001) and higher level of inflammatory markers [12]. Although this study has shown a higher association of comorbidities with PsA patients with HUC, it was one of the few articles not showing any age and sex variations among the two subtypes, and in relationship to Psout, the authors differentiated between HUC-PsA patients and persistent hyperuricemia PsA patients over follow-ups and concluded that PsA disease duration and BMI were predictor variables of persistent hyperuricemia in PsA patients. The different characteristics of patients with HUC in the selected studies are found in Table 3. In another study, it was shown that HUC occurred among one-third of the study population [13]. This study also justifies the correlation of HUC in PsA patients with cardiovascular comorbidities, diabetes, ischemic heart disease, and obesity. As a result of the multivariate analysis was shown associated with the previous comorbidities with an odds ratio of 4.95, [95% confidence intervals: 1.47; 16.67]), (3.61 [1.00; 12.98]) and (1.86 [1.04; 3.32]) respectively [13]. In a cross-sectional study conducted in Hong Kong, HUC accounted for 30.6% of the PsA population [14]. The significance of this study is that psoriatic lesions were present among 82.5% of the PsA population. Moreover, obesity was highly associated with HUC-PsA patients with an odds ratio of 4.4 (95% CI 2.0–9.5) [14]. Similar results were published in a study conducted by Widawski et al., where 30.2% of PsA patients showed HUC and (6.2%) met the 2015 ACR/ EULAR criteria for gout. In addition to these findings, patients with HUC were predominantly males and had higher ages when compared to NUC patients [15]. This study also gave a detailed characterization of HUC patients based on different aspects. For example, it showed that patients with HUC are more likely to have peripheral joint involvement and less isolated axial involvement compared to NUC (p = 0.033 and p = 0.019 respectively). It also showed that the Charlson comorbidity index (CCI) was higher for HUC patients (2.6 p = 0.005). Moreover, the multivariable analysis showed great association with male sex, high blood pressure, renal failure, and peripheral joint involvement (31). The male predominance pattern among PsA patients with HUC was also reported in another study by Galozzi et. al, where HUC was detected in 8.9% of the PsA population and reported a higher prevalence of hyperuricemia among patients with Metacarpophalangeal and wrist effusion [16]. In a population epidemiological survey research conducted in Japan, HUC was investigated as a comorbidity and was found prevalent among 13.5% of the Japanese population [17]. One study by chu et al., showed some contradicting results to all the previously mentioned studies. This study showed 26.92% of hyperuricemia among PsA patients and a high correlation of serum uric acid concentration to the level of waist circumference but demonstrated that disease activity scores are not correlated to the level of uric acid. [18].

**Table 2:** A summary of the prevalence of hyperuricemia among psoriatic arthritis patients

Article	Study design	Population	Control/comparison group	Hyperuricemia (HUC level)	Result of interest (prevalence of HUC)	Coexistence of gout	References
Al Johani et al. 2018	Prospective cohort	325 PsA patients	318 PsA with (NUC)	HUC in males > 450 µmol/l or in females > 360 µmol/l	(31.9%)	Prevalence of gout 3.5%	[12]
Gudu et al. 2017	Cross-sectional	120 PsA patients	N. A.	> 6.8 mg/dl	(27.5%)	Not investigated	[13]
Lai et al. 2018	Cross-sectional	160 PsA patients	N. A.	mean 500.7 ± 95.9 umol/L in males or 427.8 ± 83.1 umol/L in females	(30.6%)	Not investigated	[14]
Widawski et al. 2022	Case–control	242 PsA patients	169 PsA with (NUC)	≥ 360 µmol/L	(30.2%)	Prevalence of gout 6.2%	[15]
Galozzi et al. 2022	Retrospective cohort	213 PsA patients	194 PsA with (NUC)	> 6 mg/dL	(8.9%)	Prevalence based on MSU crystals	[16]
Kamiya et al. 2022	Survey (cross-sectional)	1641 PsA patients	N. A	N. A	(13.5%)	Not investigated	[17]
Chu et al. 2021	Cross sectional Observational	52 PsA patients	38 PsA with (NUC)	≥ 6mg/dL in females and ≥ 7mg/dL in male	(26.92%)	None had gout	[18]

**Table 3:** A summary of the characteristics of hyperuricemia patients in the selected studies

Article	Demographics		Disease characteristics			Laboratory/radiology variables	Associated comorbidities	Risk factors	References
	Age	Gender	Disease duration	Scales	Subtypes				
Al Johani et al. 2018	N.S	N.S	˃ duration of PsO and PsA	˃ PASI scores	N.A	˃ ESR & CRP ↑ serum creatinine ↑ liver function tests	Hypertension Angina Diabetes mellitus	Smoking Obesity	[12]
Gudu et al. 2017	N.S	N.S	N.S	N.S	N.S	Similar results	- Ischemic heart disease - Diabetes mellitus - Obesity	N.A	[13]
Galozzi et al. 2022	Older subjects	Male predominance	N.S	N.A	N.S	N.S	N.A	MCP and wrist joint effusion	[16]
Lai et al. 2018	N.S	Male predominance	No association	No association with: -Enthesitis index -TJC -SJC	N.A	No association with: -lipid profile -CRP	Overweight	N.A	[14]
Widawski et al. 2022	Older subjects	Male predominance	˃ mean age at PsA onset No difference in duration	N.A	˃ Peripheral PsA involvement ˃polyarticular disease < Isolated axial involvement	˃Radiographic destruction	Metabolic syndrome Hypertension Ischemic stroke Moderate or chronic renal failure Diabetes mellitus	˃ BMI High blood pressure	[15]
Chu et al. 2021	N.S	N.S	N.A	No association with: -TJC -SJC -VAS -PASI scores	N.A	N.A	Hypertension Hyperlipidemia	> Waist circumference	[18]

*PsA* psoriatic arthritis, *PsO* psoriasis, *ESR* erythrocyte sedimentation rate, *CRP* C-Reactive protein, *PASI scores* Psoriatic area and severity index scores, *MCP* metacarpophalangeal, *VAS* visual analog scale, *TJC* tender joint count, *SJC* swollen joint count, > greater, ↑ increase, *N.S*. non-significant, *N.A.* non-applicable

### Detection of urate crystals in synovial fluid and patients’ characteristics 

Monosodium urate (MSU) crystals are a major trigger of inflammatory processes as discussed in the pathophysiology section. Although HUC is seen as the main risk for gout development, MSU crystals are seen as the key feature in gout flares [19]. MSU crystals can be detected in the second stage of gout along with asymptomatic HUC [20]. The summary of the findings is summarized in Table 4. In a previously reported study by Galozzi et. al, MSU crystals were suggested as a better trigger for Psout than hyperuricemia [16]. Another study by the same authors highlighted the possible association between crystallopathies and other types of arthritis. They first studied the prevalence of MSU crystals among 7 categories of arthritis and showed that gout and PsA had the highest prevalence with 83.3% and 10% respectively [21]. A similar study investigated the prevalence of crystals among 10 categories of arthritis and found that the prevalence of MSU crystals was the highest in gout (97.96%) and PsA (3.34%) [22]. Other studies showed a higher prevalence of MSU crystals (68.58%) and more association of urate crystal-positive PsA patients with obesity, diabetes, ischemic heart disease, and dyslipidemia [23]. Moreover, this case–control study showed the association of synovial fluid crystals with psoriatic arthritis disease activity (OR = 15.96, 95%; CI 5.76–44.23) and no differences in age and sex distribution between PsA patients with and without urate crystals.[23].

**Table 4:** A summary of selected studies assessing the prevalence of urate crystals among arthritis and PsA patients

Article	Study design	Population	Control/comparison group	Technique	Result of interest	References
Galozzi et al. 2022	Retrospective cohort	213 PsA patients	194 PsA with NUC	Synovial fluid analysis	Prevalence of MSU crystals (2.45%)	[16]
Galozzi et al. 2016	Case–control	126 arthritis patients; 21 PsA	Arthritis patients without crystals	Synovial fluid analysis	Prevalence of MSU crystals (10%)	[21]
Oliviero et al. 2013	Case–control	2370 arthritis patients; 209 PsA	Arthritis patients without crystals	Synovial fluid analysis	Prevalence of MSU crystals (3.34%)	[22]
Genova-Popova et al. 2022	Case–control	156 PsA patient	50 Gonarthrosis	Synovial fluid analysis	Prevalence of MSU crystals (67.58%)	[23]

*PsA* psoriatic arthritis, *NUC* normal uricemic, *MSU* monosodium urate.

### The coexistence of gout and psoriatic arthritis

Studies that show the coexistence of PsA and gout (Psout) is rare and most of the cases are reported in the form of individual case reports. In other cases, incidences of misdiagnosis of gout with PsA and vice versa have been reported. For instance, Le Goff et al. reported 2 cases of gout misdiagnosed for severe PsA [24]. Moreover, this case report showed gouty ultra-sonographic appearances like snowstorms and double contour emphasis on radio signs, which shows the interplay between the different features of these two diseases. However, similar results were identified in a cross-sectional study by Antony et al. where the prevalence of gout in PsA patients was 11.4% and proved the characteristic radiological signs of gout in 12 patients [10]. Two case reports reported the coexistence of PsO, PsA, and gout in male Caucasian patients in their 60 s [25, 26]. What is significant is that in both cases gout features appeared after the diagnosis with PsA and with later stages.

Studies that directly investigated the prevalence of gout among PsA patients as the main population are scarce. In a Taiwanese nationwide population study, it was found that the prevalence of psoriatic arthritis among the gout population was 0.3% [27]. This study also investigated the association of gout with PsA and reported that gout is significantly associated with PsA with an odds ratio of (2.5, 95%) [27].

### Pathophysiology of Psout

It is known that the initiation of crystallization and deposition of MSU crystals happens after long exposure to increased serum uric acid levels [28]. Recent research has been trying to investigate the interplay between PsA, PsO, and gout starting from a common feature observed in the tripod condition, which is HUC. The cause of HUC was linked to different mechanisms: Those that increase serum uric acid or others that decrease uric acid excretion [8]. The role of urate crystals is currently being investigated as the interplay cause, especially after it was portrayed as a known danger-associated molecular patterns (DAMPs) molecule [29, 30].

**Fig. 2:**
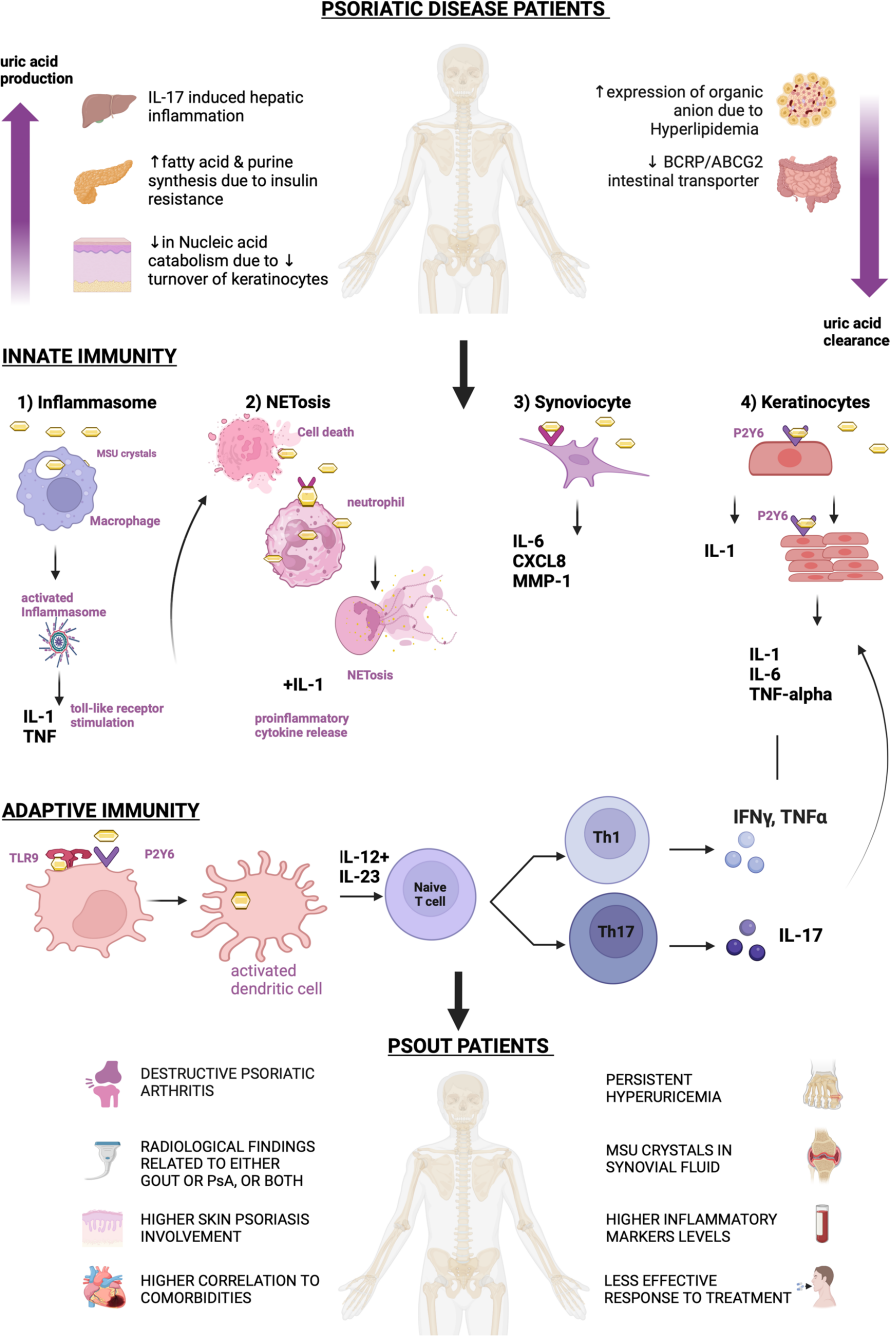
Overview of the pathophysiology of the interplay between PsA and Psout. Created with Biorender.com. *BCRP/ABCG*2 breast cancer resistance protein/ATP-binding cassette superfamily G member 2, *MSU* Monosodium urate, *IL-*1 Interleukin 1, *TNF* tumor necrosis factor, *TLR*9 Tall-like receptor 9, *P*2*Y*6 P2Y purinoceptor 6, *IL*-6 Interleukin 6, *CXCL*8 Interleukin 8, *MMP*-1 matrix metalloproteinase 1, *TNF-alpha* Tumor necrosis factor-alpha, *IL*-17 Interleukin 17, *Th*1 Type 1 T helper, *Th*17 Type 17 T helper

The pathophysiology of urate crystals in gout is broad, but it can be defined as the inter-bridging between adaptive and innate immunity as demonstrated in Fig. 2. Starting with innate immunity, monosodium urate crystals were shown to have the potential to activate polymorphonuclear cells, macrophages, keratinocytes, and synoviocytes [8]. Once phagocytized and incorporated, MSU crystals activate NLRP3 (NOD-like receptor protein 3) a pattern recognition receptor (PRR) that recognizes DAMPs [31]. Then, NLRP3 together with the adaptor ASC (apoptosis-associated speck-like protein), and pro-caspase 1 will form a Caspase-1 activating complex called NLRP3 inflammasome. This complex will activate the caspase-1 pathway and lead to the transformation of pro-interleukin 1ß into interleukin-1ß (IL-1ß), and trigger several autoinflammatory diseases [7, 8, 32–34] Other than inflammasome activation, MSU crystals can interact with polynuclear neutrophils (PNN) by 2 pathways: (1) inducing cellular death among these cells by stimulating toll-like receptors (2) inducing the interleukin 1 (IL-1) inflammation model through NETosis. In response to cell death and pathogens, neutrophils can initiate a different mechanism highlighted by the release of web-like traps made from modified chromatin, antimicrobial proteins, and enzymes called neutrophils extracellular traps in a process called NETosis [35, 36]. MSU has also displayed the potential of interacting with both keratinocytes and synoviocytes through the P2Y6 receptor signaling pathway, inducing the following proinflammatory mediators: interleukin 1α (IL-1α), interleukin 8 (IL-8/CXCL8), interleukin 6 (IL-6), and matrix metalloproteinase 1 (MPP-1) [7, 37, 38]. Concerning adaptive immunity, it has been shown that MSU crystals have the potential to activate dendritic cells directly and indirectly. The activated dendritic cell in turn secretes IL-23 and IL-12 and induce both T cell proliferation and differentiation to T helper 17 (Th17) and T helper 1 (Th1) [30, 39]. From the step forward, we can understand the pathophysiology behind Psout, especially with the ability of MSU crystals to interfere with the interleukin 23/17 (IL-23/IL-17) axis, the main pathological pathway in seronegative spondyloarthropathies [39]. The dendritic cells can be activated by stimulation via toll-like receptors, or through hyper-proliferated keratinocytes, and both will secrete different proinflammatory cytokines [40]. As the dendritic cells become activated, they induce T cell proliferation and differentiation to produce interleukins 17 and 1 (IL-17 and IL-1), which in turn stimulate the keratinocytes again as an amplification loop [7, 40–42]. Hyperuricemia and the persistent activation of the interleukin 23 and 17 (IL-23/IL-17) axis can result in a condition that combines the features of psoriatic disease and gout and introduces a new phenotype of the disease that is more inflammatory and destructive in nature.

### Impact of Psout on treatment and suggested approach

Several studies have investigated the role of Psout features on treatment outcomes of PsA patients. Secukinumab is a monoclonal antibody that inhibits IL-17, and its treatment potential has been studied in many clinical trials [43–48]. In a post hoc analysis by Felten et. al, HUC (serum uric acid ≥ 360 µmol/L), history of gout, and history of HUC lowering drugs on treatment with Secukinumab were further investigated. Upon sensitivity testing, this study proved the difference between patients’ characteristics like male dominance and older ages among patients with HUC. The study also concluded that earlier identification of Psout patients is crucial in implementing a proper management plan [49]. In other studies, the efficiency of PsA treatment was reduced and found poorer among PsA patients with HUC (OR 0.35, 95% CI 0.15–0.87), especially since HUC patients were associated with more peripheral destruction [15]. The following suggests that it is important to develop a customized treatment protocol for PsA patients with HUC. Till now there is only 1 published diagnostic and treatment algorithm for Psout. Widawski et. al, recommended the following criteria to be considered and dealt with cautious for proper diagnosis and treatment: (1) Assessment of the gout history, (2) Checking for uricemia ≥ 360 µmol/L, (3) Assessing response to uricemia lowering treatment, (4) Assessing response to PsA treatment, (5) Detection of MSU crystals through synovial fluid analysis and dual-energy computed tomography, and (6) Assessing the family history of gout [15]. Together with a structured with structured steps and questions, it was possible to create a coherent algorithm.

## Discussion

PsA is a heterogeneous disease that is associated with many conditions and diseases, and this heterogeneity makes the diagnosis more challenging for many physicians. This review shows the interplay and overlap between gout and PsA, as it focuses on the prevalence of gout and gout features among PsA patients. However, the features of gout are not exclusive to PsA patients as it can also be present among PsO patients due to the increased purine metabolism during epidermal cell turnover [50]. Moreover, PsA and PsO are defined by many scholars as different phenotypes of the same disease, which makes it harder for many clinicians to separate these two conditions. Also, a subset of PsO patients with or without PsA can present with HUC, and this feature was found associated with the degree of skin involvement among PsA patients [51, 52]. In our review, the study of Gudu et al. showed the absence of a correlation between HUC and moderate-severe skin psoriasis[13]. This finding was contradicted by another study that showed that PsA patients with psoriatic skin manifestation had higher serum uric acid than PsA patients without PsO [53]. This indicates that skin involvement and PsO should also be taken into consideration during the evaluation of patients with PsA, as it can potentially play a huge role in the shift of psoriatic disease patients to Psout phenotype.

Given that PsA is a condition with a multifactorial background, serum uric acid and MSU crystals are not always present. Also, the causes of HUC can be misleading and diverse. In a review by Al Johani, it was shown that metabolic syndrome was highly prevalent among PsA patients, especially with its main component obesity [54]. Some studies in our review showed that obesity was highly associated with PsA patients with HUC [13, 14]. This raises questions about the source of HUC and whether it shares a causal relationship with other conditions or is just an overlap. Another example of variability is the association of PsA with coronary artery and cardiovascular disease [55, 56]. These two factors were associated with an odds ratio of 1.19 (95% CI 1.14–1.24) for cross-sectional studies, 1.20 (95% CI 1.13–1.27) for cohort studies and 1.84 (95% CI 1.09–3.09) for case–control studies [55]. In most studies in the review, there was a male predominance among PsA with HUC patients which can suggest that HUC can be linked with the male sex. Even when comparing PsA patients, a study showed a male predominance among them with a 5.6:1 ratio [52]. This review also highlights the importance of distinguishing between the prevalence of HUC and persistent hyperuricemia among patients with PsA, which serves as a better marker for Psout rather than elevated serum levels of uric acid.

This review showed several studies confirming the prevalence of MSU crystals among PsA patients, this finding was also reported in a case report showing the formation of uric acid crystals and development of uric acid crystal nephropathy in a patient with erythroderma psoriasis [57]. For the detection of MSU crystals among PsA patients, the reviewed studies reported the use of synovial fluid analysis, which can be defined as an invasive and sensitive procedure. In most cases, it is not possible to withdraw synovial fluid from PsA patients unless they are going for an arthrocentesis or from other conditions to compare. Thus, the introduction of dual-energy Computed tomography -DECT, a noninvasive alternative for the detection of MSU crystals, is highly recommended. The use of DECT is an efficient technique in the detection of MSU crystals and can be used in modifying the treatment of arthritis patients suspected of gout [58]. In one study by Widawski et al., it was recommended to use both synovial fluid analysis and DECT for detection of gout among PsA patients [15]. The combination of efficient imaging techniques, collecting detailed medical history, and proper clinical assessment can help physicians in the future to detect the presence of Psout.

This review aimed to show the frequency and prevalence of gout and its features among PsA patients. The review included original work and selected articles of similar population samples and controls. Moreover, this article focused on PsA and excluded PsO involvement. This feature can be considered as a limitation as well, especially since the interplay between PsO and PsA should not be underestimated.

## Conclusion

The existence of Psout among PsA patients is prevalent and shows several associations with comorbidities, patients’ characteristics, and different clinical outcomes among affected subjects. The main question remains if Psout is a manifestation of metabolic disorders exacerbated in PsA or if it is a condition that can happen on its own with a direct risk factor of HUC in psoriatic disease. At this point, the development of an official protocol for a standardized definition, diagnosis, and treatment of Psout is needed. Moreover, it is also important to check the proper treatment indications for Psout patients as a distinct subset of both gout and PsA patients.

## Supplementary Information

Below is the link to the electronic supplementary material.

Supplementary file1 (PDF 767 KB)
